# Developing a real-time PCR diagnostic method for a potential threat to chrysanthemum, *Paratylenchus dianthus*

**DOI:** 10.21307/jofnem-2019-043

**Published:** 2019-08-05

**Authors:** Masanori Kawanobe, Koki Toyota, Hidehito Uchihara, Mikoto Takae

**Affiliations:** 1 Graduate School of Bio-Applications and Systems Engineering, Tokyo University of Agriculture and Technology, 2-24-16, Nakacho, Koganei, Tokyo, 184-8588, Japan; 2 Agri-RAND, 4-22-3, Sendagi, Bunkyo-ku, Tokyo, 113-0022, Japan; 3 Okinawa Flower Agricultural Cooperative Association, 1-10-1, Inanse, Urazoe, Okinawa, 901-2569, Japan

**Keywords:** chrysanthemum, diagnosis, high-throughput, host–parasitic relationship, imicyafos, *Paratylenchus dianthus*, pathogen, pin-nematode, plant-parasitic nematode, pot experiment, quantitative PCR, real-time PCR, technique

## Abstract

Chrysanthemum is a very popular flower in Japan and is known to be infected by many soil-borne plant pathogens including nematodes. A nematode survey in six chrysanthemum fields in Okinawa, Japan, found *Pratylenchus*, *Meloidogyne*, and *Paratylenchus* (*P. dianthus*). The first two genera are known as plant pathogens against chrysanthemum, however, *Paratylenchus* dianthus has not been reported previously. Chrysanthemum seedlings were grown in pots containing soil infected only with *P. dianthus* for two months in 2017 and 2018. The nematicide imicyafos was applied in triplicates to half of the pots (treated) while the other half were left without the nematicide (non-treated). Plant height and dry plant weight of the imicyafos treated plants exceeded those of the control plants. Also, single-photon avalanche diode value of chrysanthemum leaves was higher in imicyafos treated plants than in the non-treated plants at two-month after planting. The results suggest that *P. dianthus* may suppress the growth of chrysanthemum. For high-throughput nematode diagnosis, a real-time PCR primer set specific to *P. dianthus* was developed and its sensitivity to quantify *P. dianthus* was confirmed with a proper calibration curve. The calibration curve was developed in a simplified approach by using serially diluted DNA extracted from individual nematodes.

One of the most popular flowers in Japan is chrysanthemum (*Chrysanthemum morifolium* Ramat.), that is used on ornamental purposes in many occasions especially for new year’s and religious ceremonies, and even recognized as an edible plant in Japan. The total production of chrysanthemum in Japan in 2016 was 68bn yen (ca. $620m), which consisted one-third of the total cut-flower production and was the number one product of all the cut-flower categories (Statistics by the Japanese Ministry of Agriculture, Forestry and Fishery: http://www.maff.go.jp/j/tokei/kouhyou/hana_sangyo/). Chrysanthemum is damaged by many soil-borne pathogens including fungi, bacteria, and virus at each growth stage from the nursery bed to the field. Several plant-parasitic nematode species were also reported as important pathogens against chrysanthemum. *Aphelenchoides ritzemabosi* and *Pratylenchus penetrans* are well-known plant-parasitic nematode species that infest chrysanthemum (Goffart, 1930; Hesling and Wallace, 1961; Kobayashi, 1968). Other plant-parasitic nematodes suppressing chrysanthemum growth are *Meloidogyne incognita* (Hague, 1972), *Pratylenchus psudocoffeae* (Mizukubo, 1992), and *Pratylenchus kumamotoensis* (Mizukubo et al., 2007). The latest mentioned *Pratylenchus* spp. have been added to the list of parasitic nematodes on chrysanthemum more recently. While *A. ritzemabosi* penetrates into stems, leaves, and buds, and may be the primary cause of crinkled deformation and discoloration of the leaves on chrysanthemum, *Pratylenchus* spp. and *Meloidogyne* spp. enter into roots and cause root lesions resulting in stunting, yellowing of plant shoots, and galling on roots (Kobayashi, 1995). *Paratylenchus* spp. were also reported from chrysanthemum fields in Japan (Yamamoto and Toida, 1995; Iwahori et al., 2008), though damage to the crop has not been studied, yet. Of those plant-parasitic nematodes, *Pratylenchus* spp. are considered to be the most harmful (Yamamoto and Toida, 1995) in Japan. Since the beautiful appearance of the chrysanthemum flower is obviously important for the successful business in the flower market, the market value of chrysanthemum depends on height, flower size, the number of flowers, and color of leaves. Plant damage caused by plant-parasitic nematodes is crucial, even though plants may not wither or die by such damage.

In Okinawa (in a subtropical region, 1,600 km southwest of Tokyo), where chrysanthemum is one of the most important agricultural products, *P. penetrans* and *P. kumamotoensis* are known to inhabit in many chrysanthemum growing areas (Iwahori et al., 2008) and *Pratylenchus* may cause chronic damage to chrysanthemum (Kobayashi, 1995). Chemical treatments including nematicides, such as fosthiazate, are popular means to control nematode damage in Okinawa, however, nematode diagnosis in a chrysanthemum field is not quick and easy. Koyama et al. (2016) reported a low cost and high-throughput approach to quantify *Pratylenchus* spp. with the real-time PCR method by using pre-existing NEG primer for *P. penetrans* (Sato et al., 2007), and by developing other species-specific real-time PCR primer sets for *P. pseudocoffeae* and *P. kumamotoensis*. Those primer sets together with the one for *M. incognita* (Toyota et al., 2008) covered most widely known nematode species threating chrysanthemum cultivation in Okinawa. In some chrysanthemum fields, however, farmers struggled with symptoms of soil-borne pathogens which may not be the ones they faced before, according to our survey, and a quick and easy approach to diagnose the pathogen and to predict potential damage of chrysanthemum is in great demand. Furthermore, this study developed (lysate) calibration curves using DNA samples extracted from single nematodes, since developing lysate calibration curves may be a quicker approach than developing soil calibration curves. Though it may be ideal that each real-time PCR protocol contains specific calibration curves developed using DNA samples extracted from soil, developing soil calibration curve may require cumbersome process such as finding soil without the target nematode and inoculating the known number of the target nematode.

Our hypothesis was that plant-parasitic nematodes might be a potential threat on chrysanthemum. We further hypothesized that a real-time PCR method might be a strong diagnostic tool for assessing target plant-parasitic nematodes at low cost and high-throughput. To test these hypotheses, we analyzed plant-parasitic nematode species in six Okinawa’s chrysanthemum fields, in which plants did not grow well due to unknown reasons, and conducted pot experiments for testing *Paratylenchus dianthus* as a potential growth inhibitor of chrysanthemum seedlings. We then developed a real-time PCR method for diagnosing *P. dianthus* by testing specificity using in silico tools (e.g. BLAST) and by confirming PCR efficiency comparing the real-time PCR estimation of target nematodes with their counts by the conventional microscopic method. This study further aimed to test usefulness of a simplified approach to develop standard curves of nematode-species specific primer sets using serially diluted DNA extracted from individual nematodes.

## Materials and methods

### Soil sampling

Soil samples used were collected at Ch01, Ch02 and Ch03 from Uruma, Ch04, Ch05, Ch06 and Ch12 from Yomitan in the mainland Okinawa, Japan. Soil was collected from each field at 0 to 25 cm depth and at or around a base of post-harvest chrysanthemum plants with a spade, passed through a 5 mm aperture sieve and kept at room temperature until use. For Experiment 1 (a nematode survey), 1.5 kg of soil of each sampling site (Ch01–Ch06) was well mixed independently for further analysis. For Experiment 2, the soil samples (ca. 10 kg each) from each site (Ch04: the same timing of the soil collection for Experiment 1 in 2017; Ch05 and Ch12 in 2018) were also well mixed individually for the pot experiments.

### Nematode extraction from soil

Nematodes were extracted in triplicate from 20 g subsamples of the soil samples from each field using the Baermann funnel nematode extraction method (room temperature, 72 hr) followed by the sugar-flotation nematode extraction method (Jenkins, 1964; Kawanobe et al., 2014).

### DNA extraction from a single nematode

DNA was extracted following Iwahori et al. (2000) with minor modifications. A single nematode crushed with a sterilized filter paper chip (1×1 mm) was placed into a 200 µl Eppendorf tube containing 10 µl lysis buffer (10 mmol/liter Tris-HCl (pH 8.0), 0.1 mmol/liter EDTA, 10 g/liter of IGEPAL^®^ CA-630 (nonionic detergent; MP Biomedicals, Solon, OH), 100 µg/ml Proteinase K). After 1 hr in −85°C, the sample was thawed and incubated at 65°C for 1 hr to degrade the nematode’s body, and at 98°C for 10 min to inactivate proteinase K, and was then used as the DNA template (Kawanobe et al., 2015).

### DNA extraction from soil

DNA from soil was extracted following Kawanobe et al. (2015) with minor modification (10 g soil in duplicate were homogenized in a 15-ml Falcon tube with two stainless steel balls (3/8 inch in diam.) using FastPrep 24 (MP Biomedicals, Irvine, CA) at 4.5 m/sec for 60 sec twice, instead of in a ball mill (MM400, Retsch, Germany) for 2 min at a frequency of 20/sec in the original method). In the preliminary experiment, soil samples processed with FastPrep 24 showed equivalent results obtained with MM400 under the real-time PCR analysis (data not shown). The DNA solutions dissolved in 100 µl Tris-EDTA buffer (10 mmol/liter Tris-HCl, 1 mmol/liter EDTA, pH 8.0) were diluted 10-folds with RNase-free water and used for real-time PCR assay.

### Real-time PCR protocol

Real-time PCR assays were performed with a Step One Real-Time PCR System (Life Technologies, Tokyo, Japan). Final sample volumes of 10 µl contained 5 µl of Fast SYBR Green Master Mix (Life Technologies, Tokyo, Japan), 0.4 µmol/liter of each primer, 2.2 µl of RNase-free water, and 2 µl of template DNA. The manufacturer’s recommended conditions were used with slight modifications (95°C for 10 sec, 40 cycles of (95°C for 5 sec, and 62°C for 20 sec), and melting curve profiles were generated). A negative control was prepared with RNase-free water instead of the DNA template, and a positive control (a DNA template extracted from each target nematode) was also included.

### Real-time PCR primer sets

Real-time PCR assays were conducted using a primer set “Pdia” newly developed for *P. dianthus*, NEG (NEGf (5′- ATT CCG TCC GTG GTT GCT ATG-3′) and NEGr (5′-GCC GAG TGA TCC ACC GAT AAG-3′); Sato et al., 2007) for *P. penetrans*, Pkuma (PkumaF (5′-CGT GAA ACC GAT GAG ATG GAA AC-3′) and PkumaR (5′-CAA TGG GAG TGC GGA TGA ATA C-3′); Koyama et al., 2016) for *P. kumamotoensis*, and RKN (RKNf (5′-GCT GGT GTC TAA GTG TTG CTG ATA C-3′) and RKNr (5′-GAG CCT AGT GAT CCA CCG ATA AG-3′); Toyota et al., 2008) for *M. incognita*.

### Nematode survey in chrysanthemum fields in Okinawa (Experiment 1)

Nematode surveys were conducted in six chrysanthemum fields in April (Ch01–Ch05) and September (Ch06) 2017. All the fields were possibly damaged by plant-parasitic nematodes after examination by field specialists, yet, the nematode species in the fields were not known. The nematodes were classified under a stereo-microscope (SZX10, Olympus, Tokyo, Japan) based on their morphological characters and using a real-time PCR method for *P. penetrans*, *P. kumamotoensis*, and *M. incognita*. Pin-nematodes (*Paratylenchus* sp.) found in this study were identified as *P. dianthus* based on DNA sequence data (Accession numbers: LC462227-LC462228) of the ITS regions. For obtaining sequence data, DNA templates extracted from single pin-nematodes were amplified following Kawanobe et al. (2014) with slight modifications (a PCR primer set: TW81 (Joyce et al., 1994: 5′-GTT TCC GTA GGT GAA CCT GC-3′); rDNA26S (Vrain et al., 1992: 5′-TTT CAC TCG CCG TTA CTA AGG-3′)). The amplified PCR products were sequenced by a commercial sequencing service provider (FASMAC, Japan).

### Pot experiments (Experiment 2)

Chrysanthemum seedlings (cv. Okinootome, one of the commonly grown cultivars in Okinawa) of ca. 5 cm height with four leaves were used for pot experiments in 2017 and 2018. In total, 18 seedlings were used for each pot experiment. A subsample (1.2 kg) of each soil (Ch04 collected in April 2017, and Ch05 and Ch12 collected in April 2018) was put into a plastic pot (15 cm upper diameter; 10 cm bottom diameter; 15 cm height) together with imicyafos (0.35 g of Nemakick in a granular form (corresponding to its conventional dosage of 200 kg/ha), Agro-Kanesho, Tokyo, Japan, 1.5% a.i.) and 3.5 g chemical fertilizer (corresponding to N-P-K: 132-70-133 kg/ha, Kasei888, Omiya Green Service, Saitama, Japan). Pots without imicyafos were also prepared as non-treated controls. Experimental pots were prepared for each treatment in triplicates. Three chrysanthemum seedlings were transplanted to each pot and placed in the open air and watered when necessary during the two-month growth period.

After two-month growth in both 2017 and 2018 experiments, plant height of each seedling was measured. Then, the above-ground parts of seedlings were cut and dried at 70°C for longer than 72 hr, and plant dry weight (biomass) was measured after cooling down in a tightly sealed plastic bag. After removing the seedlings, the soil remaining in each pot was passed through a 5 mm aperture sieve and mixed well. Nematodes were extracted and counted based on their morphological character under a stereo-microscope (SZX10). For the pot experiments in 2018, single-photon avalanche diode (SPAD) values were also measured for leaves (the 6th to the 8th leaves from top) of each seedling using a SPAD meter (SPAD-502, Konica Minolta, Tokyo, Japan).

### Development of a real-time PCR primer set and calibration curves (Experiment 3)

A specific primer set (Pdia, Table 1) was designed based on the ITS region of rRNA gene sequences of *P. dianthus*. Sequence comparisons were performed to design a specific primer set using the sequence data (comparisons with different taxonomic relationships within the same family and with plant-parasitic nematode species frequently detected in Okinawa, Table 1) collected from GenBank (http://www.ncbi.nlm.nih.gov/).

**Table 1.** Comparison of the sequences in the positions of the specific primer set for *Paratylenchus dianthus* (Pdia) with different taxonomic relationships.

	Sequence (5′ → 3′)	
Nematode species for sequence comparison (accession no.)	Forward	Reverse
PdiaF/R	TGACTG---TCG--AAGGCATAGTGGTAGA	CGGCACCTAGAGCAA---GGTACTCA
The genus *Paratylenchus*		
*Paratylenchus aquaticus* (KF242278)	..G ...CCG .-.--.G.CGT .- .C.A ....	.......C.AGC.-----........
*P. guangzhouensis* (KT725626)	..G...ACAAT.--....GT.-........	.......AC-.-.T----........
*P. hamatus* (KF242258)	......CGT.TT--.......T........	.......AGT....C---........
*P. lepidus* (EF126178)	......TG-.T.--.CA..G.T........	..........C....---........
*P. leptos* (KR270605)	..G...-CG.T.--...CGT.-AC.TG.TT	.......AC-T-.C----........
*P. minutus* (EF126180)	..T...TG-.G.--.C.....T........	........C.C..T.---...G....
*P. nanjingensis* (KM366103)	..G..TGCAGT.--...CGT.-.......-	........C-C-.T----........
*P. nanus* (KY468906)	......CGT.TT--.......T........	.......AGT.T..C---........
*P. rostrocaudatus* (KR270604)	..G...-CG.T.--...CGT.-AC.AG..T	.......AC--..T----........
The same subfamily Paratylenchinae		
*Gracilacus bilineata* (EU247525)	..G...ACAAT.--...CGT.-........	.......AC-.-.T----........
*G. aculenta* (EU247526)	..G..TGCAAT .--...CGT .- .......-	........C-T-.T----........
The other subfamily Tylenchulinae		
*Meloidoderita kirjanovae* (DQ768427)	..G...C TGA-.--.... GT .-CC.AC. .G	..A....A..T-.T----...GT...
Plant-parasitic nematode species frequently detected in Okinawa, Japan		
*Ditylenchus destructor* (KX181647)	..G...---.-..TG.A.G.A.AC....CG	.......A.-...C.----.TG....
*Helicotylenchus dihystera* (LC030373)	..G...---C- ..TG..A .GAC.C.AC ..G	....G.AACAT..T---C..CG....
*Hoplolaimus columbus* (AB933480)	..G...---C-..TG..ATGAC.C.....G	....T.TCTAC---.---C.AG....
*Meloidogyne arenaria* (LC030354)	..G...---.ATATGT ..TGACA..... .G	....-.TC.-CTT..---.AGG....
*M. incognita* (KY985255)	..G...---.ATATGT ..TGACA. .....G	....-...CACTT..---.AGG....
*M. javanica* (AY438555)	..G...---.ATATGT..TGACA... ...G	....-.TC.-CTT..---.AGG....
*Mesocriconema xenoplax* (FN433851)	..G...---C..-TG.T.TG.T.-C.A....	......-.-...TCC---........
*Pratylenchus coffeae* (FJ712906)	..G...---.G- ATG..A ..AC.. .....G	....C.TGG..CT..TTG C.GG....
*P. kumamotoensis* (LC030317)	..G...---.A-ATG.....ACAC.....G	..T.C.AC--.T.T.---..GG....
*P. pseudocoffeae* (LC030337)	..G...---.G-ATG.....AT.C.....G	..T.C.AC .C.CA.TA AAT.GG....
*P. penetrans* (LC030333)	..GT..---- .TATC..A.. A.CG.TAG ..	....C.....-...---TT.GG....
*P. zeae* (KY424187)	..GT..---.-..TG....GACA......G	....-..GT-...CGT--.-GG....
*Rotylenchulus reniformis* (AY335191)	..G...---.-..TG..A..AC.C.....G	....G..A.ACCAG----..CG....
*Tylenchorhynchus annulatus* (MG430283)	..G...---.-..TG..ATGAC.C.....G	....-..C...-...---C.GG....
*T. leviterminalis* (AB933474)	..G...---.-..TG..ATG AC.C.....G	.A ..-..A.-..AC.-- CA.GG..AT
*T. zeae* (J461599)	..G...---.G-ATG..ATGAC.C.....G	.. ..C..-...-..---GCAGG....

Note: Hyphens indicate deletion of the corresponding base and dots indicate the same base as the primer sequence.

For generating calibration curves (lysate calibration curves), *P. dianthus* and *M. incognita* were collected from chrysanthemum field soils and DNA was extracted from a single nematode, in six replicates. Then, each 10 µl DNA solution was serially diluted (1:10, 1:40, 1:160, 1:640, 1:2560, and 1:10240, equivalent in DNA concentrations to soil DNA sample of 0.7 to 680 nematodes per 20 g soil) with RNase-free water. The diluted DNA samples were used as templates in real-time PCR assays to generate lysate calibration curves using the primer sets of Pdia and RKN. DNA was also extracted, in duplicates, from the subsample of the same soil used for nematode extraction at the end of the pot experiment (Ch12 only: the imicyafos treated and non-treated pots, in triplicates) in 2018. The DNA extracts were diluted 10-folds with RNase-free water and used for a real-time PCR assay to develop a soil calibration curve for Pdia. Lysate calibration curves for NEG (*P. penetrans*; Kawanobe et al., 2015) and Pkuma (*P. kumamotoensis*; Koyama et al., 2016) were adjusted to make the DNA dilution rate equivalent to soil calibration curves. This conversion reflected the difference in DNA concentrations between soil DNA samples (ca. 0.015 nematode in a 100 µl DNA solution, based on the extraction from 0.25 g oven-dried soil (equivalent to 0.29 g fresh soil with 15% water content) out of 20 g fresh soil containing one nematode) and single nematode DNA samples (one nematode in a 10 µl DNA solution).

### Application of real-time PCR method to field nematode survey (Experiment 4)

DNA was extracted, in duplicates, from the subsample of the same soil sample used for the nematode extraction of the nematode survey (Ch01–Ch06). After extraction, DNA samples were diluted 10-folds with RNase-free water and used as templates. Specific primer sets for *P. dianthus* (Pdia), *P. penetrans* (NEG), *P. kumamotoensis* (Pkuma), and *M. incognita* (RKN) were used for real-time PCR assays. Soil calibration curves were applied for quantifying *P. dianthus* and *M. incognita*, and so were lysate calibration curves for all the nematode species. Nematode density of each plant-parasitic nematode species was estimated using each calibration curve by applying the C_q_ (quantification cycle) value measured in the real-time PCR assay.

### Statistical analysis

The statistical difference was determined by Student’s *t*-test comparing control and test groups or analyzed by ANOVA followed by Tukey–Kramer’s test for multiple comparisons. Analysis of covariance (ANCOVA) was used to test the difference between the two regression slopes and intercepts following Ichihara (1990). Statistical analyses were conducted using Microsoft Excel and its add-in software Statcel (3rd ed., OMS, Tokyo, Japan).

## Results

### Nematode survey (Experiment 1)

The survey of the six locations revealed four different plant-parasitic nematode species (Table 2). *P. penetrans* was detected only in Ch01, while *P. kumamotoensis* was found in Ch02 and Ch03. *P. dianthus* was the most widely distributed species found in four locations (Ch02–Ch05). *M. incognita* was only present in Ch06.

**Table 2.** Nematode (per 20 g soil) survey using microscopic method in chrysanthemum fields and nematode estimation (per 20 g soil) using real-time PCR method.

	Microscopic method				Real-time PCR estimation					
Sample^a^	Nematode species				Primer and calibration curve^b^					
					NEG^c^		Pkuma^d^	Pdia^e^		RKN^f^
	*Pratylenchus penetrans*	*P. kumamo- toensis*	*Paratylenchus dianthus*	*Meloidogyne* sp.	Soil	Lysate	Lysate	Soil	Lysate	Lysate
Ch01	151	nd	nd	nd	151	110	nd	nd	nd	nd
Ch02	nd	7	10	nd	nd	nd	8	30	40	nd
Ch03	nd	22	47	nd	nd	nd	42	219	240	nd
Ch04	nd	nd	119	nd	nd	nd	nd	595	589	nd
Ch05	nd	nd	38	nd	nd	nd	nd	324	341	nd
Ch06	nd	nd	nd	315	nd	nd	nd	13	19	731

Notes: nd indicates non-detected. ^a^Ch01: 26° 22′ 12′′N 127° 50′ 21′′E, Ch02: 26° 22′ 19′′N 127° 50′ 02′′E, Ch03: 26° 22′ 15′′N 127° 50′ 00′′E, Ch04: 26° 25′ 02′′N 127° 43′ 07′′E, Ch05: 26° 24′ 52′′N 127° 43′ 05′′E, Ch06: 26° 22′ 12′′N 127° 50′ 21′′E; ^b^Soil and lysate are based on the calibration curves and primer sets developed in each primer set; ^c^ Sato et al. (2007) (primer) and Kawanobe et al. (2015) (calibration curve) for *Pratylenchus penetrans*; ^d^ Koyama et al. (2016) for *P. kumamotoensis*; ^e^this study for Paratylenchus dianthus; ^f^ Toyota et al. (2008) (primer) and this study (calibration curve) for *Meloidogyne* sp.

### Plant growth and pin-nematode density after two month (Experiment 2)

**Figure 1:**
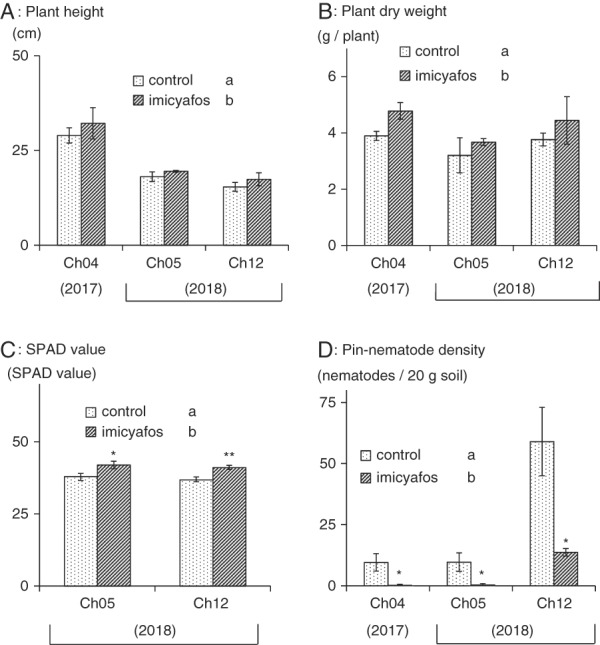
Results of pot experiments. A: Plant height; B: Plant dry weight of above-ground parts; and C: SPAD value (only for Ch05 and Ch12); and D: Pin-nematode densities with soil treated with imicyafos (3 kg a.i./ha), or non-treated as a control in triplicates, after 2-month growth period. Asterisks indicate significant difference from a control group by Student’s *t*-test (*p < 0.05 and **p < 0.01). Different alphabet letters indicate significant difference by a multiple comparison (Tukey–Kramer’s test, main effect (treatment): *p* < 0.05; interaction effect (treatment × soil: *p* > 0.05) following two-way ANOVA (alphabet letters in Figure 1D are only for reference; interaction effect (treatment × soil): *p* < 0.05).

Plant height of chrysanthemum seedlings grown with imicyafos was significantly (a multiple comparison with the Tukey–Kramer’s test, main effect (treatment): *p* < 0.05; no interaction effect (treatment × soil): *p* > 0.05) higher than of the non-treated seedlings after two-month growth (Fig. 1A). Dry weight of the imicyafos treated seedlings was also significantly (main effect (treatment): *p* < 0.01; no interaction effect (treatment × soil): *p* > 0.05)) higher than of the non-treated seedlings (Fig. 1B). SPAD value of imicyafos treated seedlings for both Ch05 and Ch12 in 2018 was significantly (main effect (treatment): *p* < 0.01; no interaction effect (treatment × soil): *p* > 0.05) higher than of the non-treated seedlings (Fig. 1C). *P. dianthus* densities of imicyafos treated seedlings were significantly (Student’s *t*-test: *p* < 0.05) lower than of the non-treated seedlings in all the three soils (Fig. 1D). A multiple comparison showed significantly different *P. dianthus* densities between the imicyafos treated and non-treated seedlings, yet the result should be treated as reference only due to the existence of interaction effect (main effect (treatment): *p* < 0.01; interaction effect (treatment × soil): *p* < 0.05). No other plant-parasitic nematodes were observed in all soil samples.

### Development of calibration curves (Experiment 3)

**Figure 2:**
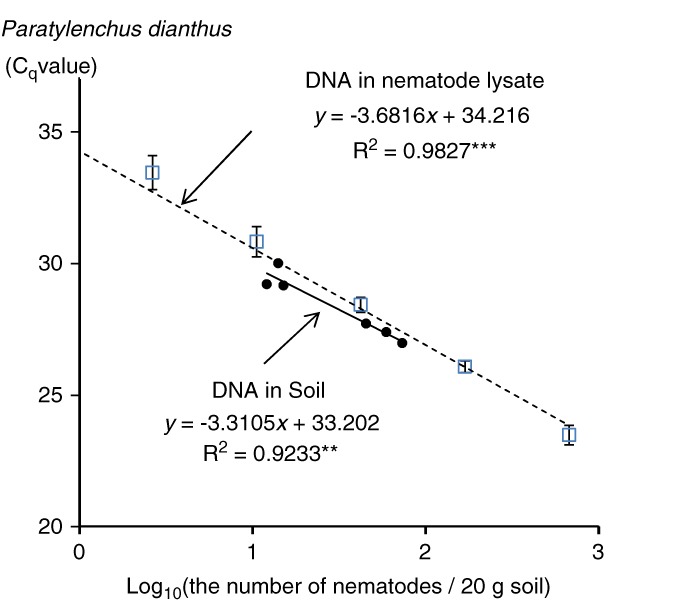
Relationship between calibration curves of soil DNA samples and nematode lysate for *Paratylenchus dianthus*. Solid line: a calibration curve derived from DNA samples extracted from Ch12 experiment at two-month after planting. Dotted line: a calibration curve derived from DNA samples extracted from nematode lysate. The curve was related to the DNA concentrations equivalent to the ones obtained by using DNA samples from soils. ***p < 0.001 and **p < 0.01.

The calibration curves for *P. dianthus* and *M. incognita* developed by serially diluted DNA templates from single nematode lysate (*y*-axis: C_q_ values, and *x*-axis: log_10_ (the number of nematodes equivalent per 20 g soil) were *y* = − 3.6816*x* + 34.216 (*R* ^*2*^ = 0.9827, *p* < 0.001, Fig. 2) and *y* = − 3.4585*x* + 33.798 (*R* ^*2*^ = 0.9985, *P* < 0.001, Table 3), respectively. The calibration curve for *P. dianthus* developed using DNA extracted from soil (*y*-axis: C_q_ values, and *x*-axis: log_10_ (the number of nematodes per 20 g soil) was *y* = −3.3105*x* + 33.202 (*R* ^*2*^ = 0.9233, *p* < 0.01, Fig. 2). The slopes and the intercepts of the calibration curves were not different (ANCOVA, *p* = 0.05) between calibration curves using serially diluted DNA and soil DNA for each of *P. dianthus* and *P. penetrans* (Table 3), respectively.

**Table 3.** Calibration curves to estimate nematodes in soil.

		Calibration curve		
Nematode species	Primer set	Type^a^	Equation	Reference
*Pratylenchus penetrans*	NEG	Soil	*y* = −3.1449*x* + 34.834 (*R* ^2^ = 0.97***)	Kawanobe et al. (2015)
		Lysate	*y* = −3.3664*x* + 34.858 (*R* ^2^ = 0.9019***)	
*P. kumamotoensis*	Pkuma	Lysate	*y* = −3.2173*x* + 34.178 (*R*² = 0.9987***)	Koyama et al. (2016)
*Meloidogyne incognita*	RKN	Lysate	*y* = −3.4585*x* + 33.798 (*R*² = 0.9985***)	This study

Notes: ^a^Soil: developed using DNA extracted from soil, lysate: developed using DNA extracted from nematode lysate. *y*: C_q_ value and *x*: log_10_ (the number of nematodes per 20 g soil) or equivalent. *p<0.05; **p<0.01; ***p<0.001.

### Estimation of nematodes using real-time PCR method (Experiment 4)

All the real-time PCR assays using nematode species-specific primer sets detected the relevant nematode species, which were recorded from the nematode survey using the Baermann and sugar-flotation methods (Table 2). The estimated numbers of each nematode species were equivalent to or exceeded (×0.7–×9.0) those in the nematode survey with the microscopic method. The real-time PCR approach was also sensitive to detect *P. dianthus* in Ch06 where the microscopic approach did not detect *P. dianthus*.

## Discussion

Four plant-parasitic nematode species, *P. penetrans, P. kumamotoensis*, *P. dianthus*, and *M. incognita* were detected in the field surveys. Even though the survey was conducted in only six chrysanthemum fields within 20 km distance, the most commonly found plant-parasitic nematode species (*Pratylenchus* spp. and *Paratylenchus* sp.) on chrysanthemum in Okinawa were detected in this survey and the result obtained was consistent with Iwahori et al. (2008). *Pratylenchus* and *Meloidogyne* were reported as pathogens on chrysanthemum in Japan (e.g., Saigusa and Matsumoto, 1961; Kobayashi, 1995), however, *P. dianthus* was not recognized as a plant pathogen on chrysanthemum in the previous studies. *P. dianthus* was described and reported to cause poor growth of green-house carnations (Jenkins and Taylor, 1956), and was also found to cause severe leaf drop of *Euonymus japonicus*, an evergreen shrub (Liu, 1995).

The nematicide imicyafos suppressed the population density of *P. dianthus* in all the pot experiments. The growth indicators, plant height, plant dry weight, and SPAD value, of the pot experiments at two-month after planting indicated that *P. dianthus* may have inhibited growth of chrysanthemum seedlings. At one-month after planting, plant height was not different between the imicyafos treatments and the control (data not shown). Yet, the difference became clear and the average plant height of the imicyafos treatment in each experiment was higher than that in the control at two-month after planting. The leaf color started to be gradually different after one-month, and showed obvious difference at two-month after planting. Though no severe leaf drop or stunting was observed, ratios of yellowish leaves were clearly lower in the imicyafos treatment than in the control at two-month after planting. The overall results of the experiments suggested that *P. dianthus* may suppress the growth of chrysanthemum during the two-month growth period. The exact mechanism of the damage caused by *P. dianthus* is yet to be described.

Many of the chrysanthemum fields in the Kyushu-Okinawa region were infested with the root-lesion nematodes *Pratylenchus* spp. (Uesugi et al., 2009), which damaged chrysanthemum plants (Kobayashi, 1995) and were the main targets of nematode control in chrysanthemum fields. This study suggested that the pin-nematode *P. dianthus* may also need to be controlled to avoid plant growth inhibition. Imicyafos may not alter the soil microbial and nematode community structures (Wada and Toyota, 2008; Wada et al., 2011). The possibility might not be denied in this study that damage is caused by other potential pathogens such as fungi and bacteria and *P. dianthus* plays a role as a vector. To address this lacking information, further studies are needed to confirm the exact process of plant damage.

Species-specific primer set was developed for a real-time PCR method to quantify *P. dianthus* and had eight or more mismatches and/or gaps in the primer regions against nematode species in the same genus. In the same subfamily and the same family, 16 to 22 mismatches and/or gaps were found. For the other plant-parasitic nematode species frequently found in Okinawa, more than 20 mismatches and/or gaps were found, including more than 9 mismatches and/or gaps within 10 bp from 3′ end of both primers. Taking these results into consideration, the primer set Pdia may be species specific enough to be applied to DNA sample collected in Okinawa. Previous studies showed the importance of mismatch on the position closer to the 3′ end of the primer (Bru et al., 2008; Isenbarger et al., 2008; Lee et al., 2008, Kawanobe et al., 2015; Koyama et al., 2016). The primer set Pdia had one mismatch to *Paratylenchus hamatus* and *Paratylenchus nanus* within 10 bp from the 3′ end of both primers, so did one mismatch PdiaR to *Paratylenchus lepidus* within 10 bp from the 3′ end. Kawanobe et al. (2015) discussed that more than two mismatches within ten bases from the 3′ end resulted in reducing amplification efficiency to less than 1% and Koyama et al. (2016) showed that one mismatch within 10 bp from the 3′ end of both primers decreased the amplification efficiency to 4.7%. These suggested that Pdia might amplify these three *Paratylenchus* spp. at an amplification efficiency of less than 4.7%. Since *P. lepidus* was reported from tea plantations in Taiwan (near from Okinawa; Chen et al., 2007), it may be important to know the possible misidentification under the real-time PCR method.

The real-time PCR assays using the primer sets, NEG, Pkuma, Pdia, and RKN properly quantified targeted nematode species and were more sensitive or equivalent to the nematode survey results obtained with the microscopic method. The results of real-time PCR quantification using soil calibration curves for NEG and Pdia were equally or more sensitive than those of the survey, since in Ch06, *P. dianthus* was detected by the real-time PCR method, but not the microscopic approach. In addition, nematode densities estimated using nematode-lysate calibration curves for all the four primer sets NEG, Pkuma, Pdia, and RKN were also equally or more abundant than those of the survey. It is one of the advantages of real-time PCR approach that may detect nematodes in dormant state, which might be impossible to extract from soil using conventional Baermann nematode extraction method (Min et al., 2012), and in an egg form, which might be difficult even with the sugar-flotation method. The most important advantage to use a nematode-lysate (just using single nematodes and serially dilute the templates) calibration curve under the real-time PCR method is its simple and easy process to develop the curve comparing to a soil DNA calibration curve, which requires preparing soil samples with different numbers of target nematodes. By taking due care, the real-time PCR method using a nematode-lysate may be feasible, low-cost, and high-throughput approach for nematode diagnosis.

As a conclusion, this study revealed that *P. dianthus* may inhibit growth of chrysanthemum in Okinawa, and developed a high-throughput method to quantify *P. dianthus* using newly developed Pdia primer set and calibration curves under the real-time PCR approach.
